# Retraction: Assessment of seasonal, substrate and hormonal factors influencing the propagation and rooting of *Camellia rubriflora's* cuttings

**DOI:** 10.1242/bio.062662

**Published:** 2026-06-24

**Authors:** Tinh Thi Nguyen, Vinh Thi Pham, Huong Thi Nguyen, Hoai Thi Duong, Cuong Manh Duong

The journal is retracting *Biology Open* (2026), bio.062383 (doi:10.1242/bio.062383) due to duplicate publication.

Following publication of the accepted manuscript, the authors informed the journal that research described in the manuscript had previously been published in the Science Journal of Agriculture and Environment (Tạp chí Nông nghiệp và Môi trường; pISSN 3093-3382) in November 2025 [Nguyễn, T. T., Dương, T. T. H., Nguyễn, T. T. H., Bùi, Đ. L., Nông, T. T. and Ngô, X. B. (2025). Nhân giống cây trà hoa đỏ cánh đơn (Camellia rubriflora) bằng phương pháp giâm. *Tạp chí Nông nghiệp và Môi trường*, Số Tháng 11, 69-77].

The authors did not declare this duplicate submission at the time of submitting the manuscript to Biology Open.

Tinh Thi Nguyen and Hoai Thi Duong have not explicitly stated whether they agree with this retraction. Vinh Thi Pham, Huong Thi Nguyen and Cuong Manh Duong agree with this retraction.

